# Microwave Effect in Hydrolysis of Levoglucosan with a Solid Acid Catalyst for Pyrolysis‐Based Cellulose Saccharification

**DOI:** 10.1002/open.202300311

**Published:** 2024-05-29

**Authors:** Takashi Nomura, Eiji Minami, Haruo Kawamoto

**Affiliations:** ^1^ Graduate School of Energy Science Kyoto University Yoshida-honmachi, Sakyo-ku, Kyoto 606-8501 Japan

**Keywords:** levoglucosan, hydrolysis of anhydrous sugar, solid acid catalyst, microwave-assisted chemistry, selective heating

## Abstract

Pyrolysis‐based saccharification consisting of fast pyrolysis followed by hydrolysis of the resulting anhydrosugars such as levoglucosan is a promising method for converting cellulosic biomass into glucose that can be used for producing biofuels and biochemicals. In the present study, hydrolysis of levoglucosan was evaluated in water with a polystyrene sulfonic acid resin (a solid acid catalyst) by heating under microwave irradiation or in an oil bath at 95 °C–120 °C. When the equilibrium temperature of the solution was the same, the conversion rate of levoglucosan was greater under microwave irradiation than in an oil bath. Model experiments indicate that the sulfonyl groups of the solid acid catalyst were selectively heated by microwave irradiation. The temperature of the reaction solution in the vicinity of the catalyst was locally higher than the equilibrium temperature of the solution, which enabled hydrolysis to proceed efficiently.

## Graphical abstract

The hydrolysis of levoglucosan was carried out in water using a solid acid catalyst under microwave irradiation for pyrolysis‐based saccharification of cellulose and pulp. The sulfonyl groups of the solid acid catalyst were selectively heated by microwave adsorbing, resulting in more efficient hydrolysis than using a conventional oil bath when the equilibrium temperature of the solution was the same.

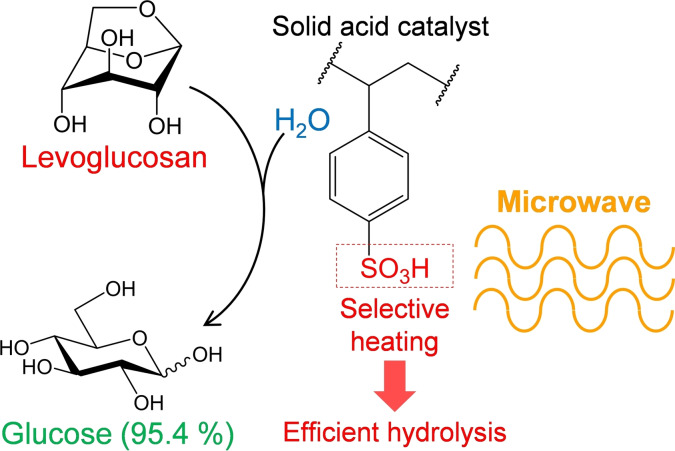

## Introduction

Cellulose is a renewable resource that can be used to produce biofuels and biochemicals. Glucose, obtained by saccharification of cellulose, can be converted by fermentation into various chemical feedstocks such as ethanol, acetic acid, and lactic acid. Currently, glucose is produced from edible sugars and starches. However, because using agricultural products as raw materials competes with their use as food, producing glucose from inedible cellulose is highly desirable.

Various methods for cellulose saccharification have been proposed, including acid hydrolysis,^[1]^ enzymatic hydrolysis,^[2]^ and hydrothermal treatment.^[3]^ However, because crystalline cellulose is physically and chemically stable, severe reaction conditions are required for hydrolysis. For example, conventional acid hydrolysis involves solubilizing cellulose with a large quantity of concentrated sulfuric acid, which is then diluted with water and heat‐treated at 120 °C to yield glucose.^[4]^ This process requires a large quantity of base for neutralization and much energy to remove the added water.

Fast pyrolysis under dry conditions is an efficient method for decomposing crystalline cellulose to produce high yields (maximum 60–70 wt% on cellulose) of levoglucosan (LG, 1,6‐anhydro‐β‐D‐glucopyranose), which can be hydrolyzed into glucose under mild conditions.^[5]^ By minimizing the quantity of water added during hydrolysis, highly concentrated glucose solutions can be easily obtained. Therefore, pyrolysis‐based saccharification which consists of fast pyrolysis of cellulose followed by hydrolysis of the resulting LG and 1,6‐anhydro‐β‐D‐glucofuranose (furanose‐type anhydrous sugar of glucose) might be a promising method for cellulose saccharification.

Previous studies have mainly used sulfuric acid for hydrolyzing LG,^[6]^ but doing so has drawbacks such as corrosion of the reaction tube as well as difficulty in catalyst recovery and neutralization. Solid acid catalysts are preferable by comparison because they have little impact on the reaction tube and can be easily separated from the product by filtration and reused. However, hydrolyzing LG by using solid acid catalysts has rarely been reported.^[7]^

Microwave heating has the advantage of selectively heating certain substances based on their absorption properties. Previous studies reported that solid catalysts made of activated carbon or zeolite were selectively heated with microwaves, resulting in faster reaction rates than conventional heating at the same equilibrium temperature of the liquid phase.[^8^, ^9^, ^10^] Hydrolysis of cellulose with activated carbon‐based acid catalysts under microwave irradiation has also been reported.[^8^, ^10^] However, since cellulose is insoluble in water, the reaction conditions are different from those of hydrolysis of LG. In addition, the microwave effects on the solid catalysts are not clearly explained in terms of the roles of active site and carbon support for selective heating. The hydrolysis of LG must occur near the acidic sites. In the present study, the hydrolysis of LG was investigated by using a polystyrene‐based sulfuric acid catalyst under microwave irradiation. Model experiments were also conducted to understand the role of the sulfuric acid group of the catalyst. Finally, the hydrolysis mechanism under microwave irradiation is discussed.

## Materials and Methods

Amberlyst 15 (ORGANO, Tokyo, Japan), a polystyrene‐based ion exchange resin with a sulfonic group, was used as a solid acid catalyst. Zhou et al.^[11]^ reported its properties, including an acidity of 4.70 mmol/g, an average pore diameter of 32 nm, a particle size of 90–200 μm, and a maximum operating temperature of 120 °C. A 10 mL sealed vessel containing 0.6 mL of the solid acid catalyst and 2.4 mL of an aqueous solution of LG (Tokyo Chemical Industry, Tokyo, Japan; purity>99.0 %) was used for hydrolysis [LG concentration: 730 mg/L (water)].

**Figure 1:**
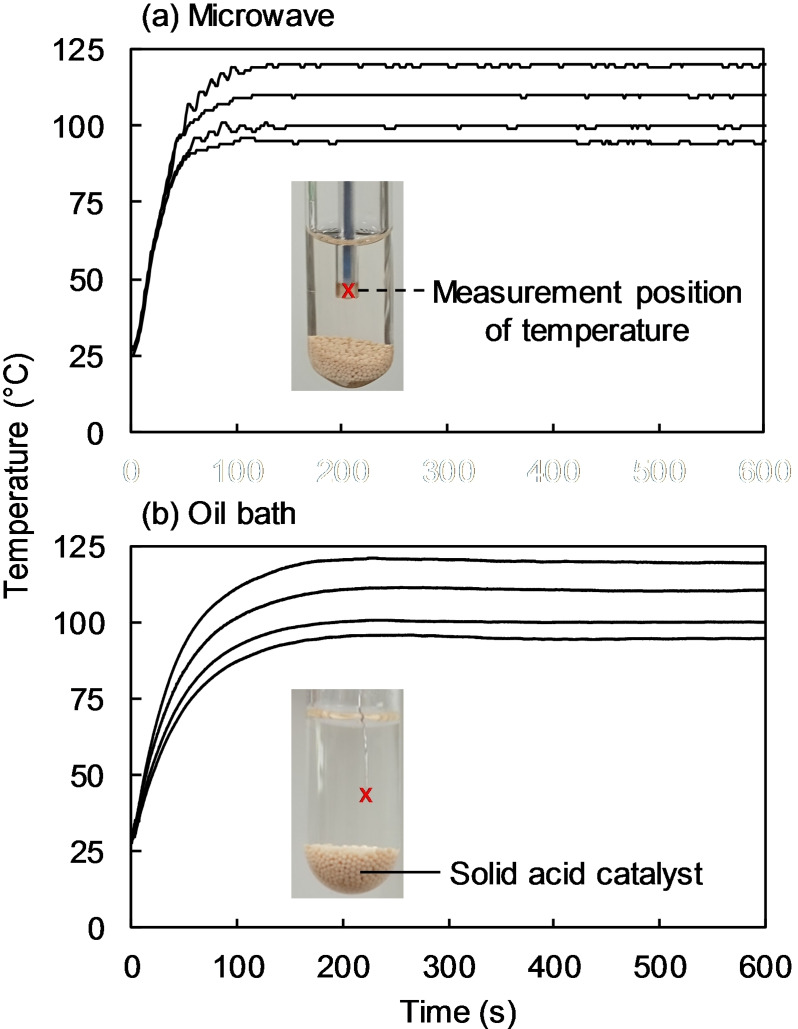
Measurement position of temperature (×) and temperature profiles in (a) microwave heating and (b) oil bath heating.

Microwave heating was performed with a single‐mode microwave synthesizer (Discover SP, CEM, Matthews, NC, USA). The maximum power set for the microwave was 50 W and the frequency was 2455 MHz. The system included a sealed vessel designed to maintain the internal pressure below the maximum pressure (1.7 MPa) by leaking the gaseous portion when the pressure exceeds the maximum. Thus, water can be heated to 200 °C with this system. The reaction temperature (95 °C, 100 °C, 110 °C, and 120 °C) was controlled by monitoring the temperature with a fiber optic thermometer at the location marked with a red “x” in Figure 1a. Oil bath heating was also performed [Figure 1(b)], and the temperature of the reaction mixture was monitored at the same location. In both cases, the reaction time was counted, starting after the temperature of the reaction mixture reached the set temperature.

After treatment for the specified time, the reaction mixture was filtered and the filtrate was subjected to glucose analysis. The yield of glucose (mol% based on LG) was determined by high‐performance liquid chromatography, which was performed with a Prominence chromatograph (Shimadzu, Kyoto, Japan) by using a CarboPacTM PA1 column (4 mm×250 mm, Thermo Scientific, Waltham, MA, USA) at 35 °C, with 0.03 M NaOH as the eluent and a flow rate of 1.0 mL/min. An electrochemical detector (DECADE Elite, Antec Scientific, Zoeterwoude, the Netherlands) was used.

## Results and Discussion

**Figure 2:**
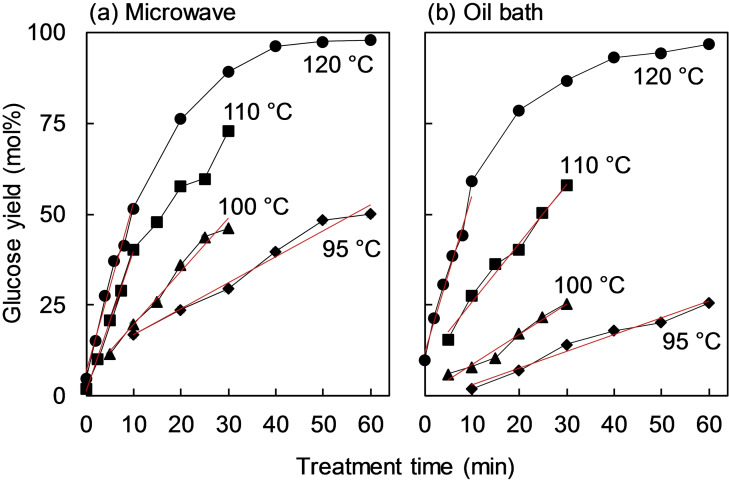
Glucose yield after hydrolysis of LG with a solid acid catalyst (Amberlyst 15) at 95 °C to 120 °C in (a) microwave heating and (b) oil bath heating.

Figure 2 shows the glucose yields obtained from aqueous LG solutions treated with the solid acid catalysts under microwave heating [Figure 2(a)] and oil bath heating [Figure 2(b)]. In both methods, LG was selectively hydrolyzed into glucose. The glucose yield from levoglucosan reached nearly 100 mol% after heating at 120 °C for 60 min under both microwave heating and oil bath heating, demonstrating the effectiveness of the solid acid catalyst (Amberlyst 15) for hydrolyzing LG.

The glucose yield was higher under microwave heating than in the oil bath, especially at the relatively low temperatures of 95 °C (29.5 and 14.2 mol%), 100 °C (46.2 and 25.5 mol%), and 110 °C (72.9 and 58.0 mol%; values in parentheses: yields after 30 min under microwave irradiation and in the oil bath, respectively); but the yields at 120 °C were similar (89.1 and 86.7 mol%, respectively).

**Figure 3:**
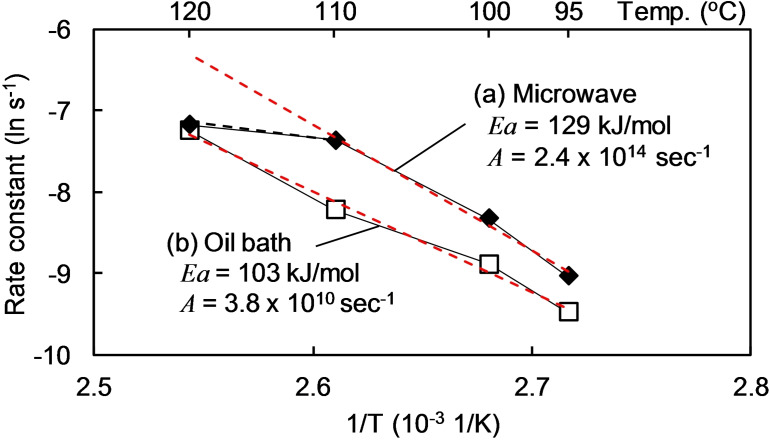
Arrhenius plots for rate constants of levoglucosan hydrolysis with the solid acid catalyst (Amberlyst 15) (microwave heating and oil bath heating).

To understand the microwave effects on the hydrolysis of LG with the solid catalyst, the hydrolysis rate constants were estimated by assuming a pseudo‐first order reaction. The reaction rates were evaluated from the slopes of the linear portion at the initial stage of the reaction (red lines in Figure 2). Figure 3 shows the corresponding Arrhenius plots with activation energies [*E*a (kJ/mol)] and pre‐exponential factors [*A* (s^−1^)], both of which were obtained from equation 1:

(1) lnk=-(Ea/R)(1/T)+lnA

where *k* is the reaction rate constant, *Ea* is the activation energy (kJ mol^−1^), *R* is the molar constant of the gas (8.3143 J mol^−1^ K^−1^), *T* is the absolute temperature (K), and *A* is the pre‐exponential factor (s^−1^).

The Arrhenius plot (oil bath) was approximated by using a straight line with an *E*a 103 kJ/mol and *A* 3.8×10^10^ s^−1^. In contrast, the microwave plot had an inflection point at 110 °C, suggesting that the reaction mechanism changed around this temperature. The rate constants were similar for both heating conditions at this temperature. This is in good agreement with the aforementioned description of the yield of LG. Based on the straight line at the lower temperatures, *E*a 129 kJ/mol and *A* 2.4×10^14^ s^−1^ were obtained. Consequently, the values of *E*a and *A* were larger under microwave heating than in the oil bath.

**Figure 4:**
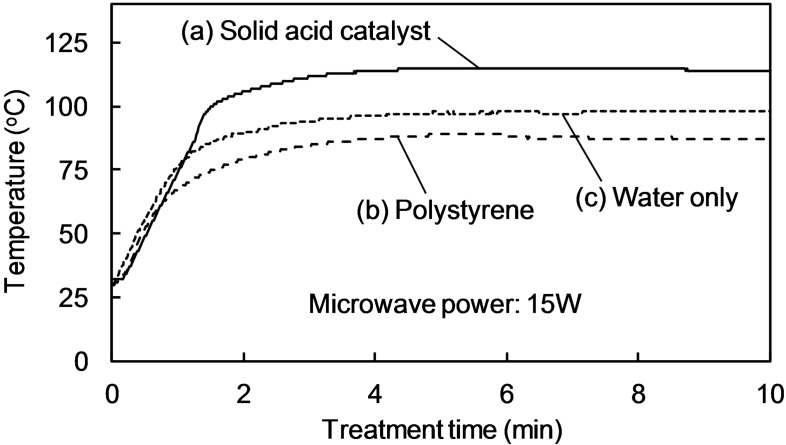
Solution temperature profiles under constant microwave power (15 W) in (a) solid acid catalyst (Amberlyst 15) and water, (b) polystyrene and water, and (c) water only.

To study the microwave effect in more detail, the temperature profiles were measured for various mixtures: solid acid catalyst in distilled water [Figure 4(a)], polystyrene in distilled water [Figure 4(b)], and distilled water only [Figure 4(c)]. Each mixture was irradiated with microwaves at a constant power of 15 W. The temperature increased and reached a constant value when the microwave heating was equilibrated with the heat dissipation from the surface of the reaction vessel.

The equilibrium temperature of polystyrene in water [Figure 4(b), 86 °C] was slightly lower than that of water alone [Figure 4(c), 98 °C], indicating that the microwave absorptivity of polystyrene was lower than that of water. However, the solid acid catalyst in water [Figure 4(a)] had the highest equilibrium temperature of ca. 120 °C. The solid acid catalyst (Amberlyst 15) has a sulfonyl group (SO_3_H) attached to the polystyrene benzene ring. Therefore, the difference between Figure 4(a) and 4(b) was attributed to the temperature increase because of microwave absorption of the sulfonyl group. These results indicate that the sulfonyl group of the solid acid catalyst was selectively heated by microwave irradiation, as discussed next.

Microwave heating of sulfonated activated carbon catalysts has been reported.[^8^, ^10^] However, these papers do not discuss the specific effects of using microwaves. The current study demonstrates that the sulfonyl groups in the polystyrene‐based catalysts were selectively heated by microwave irradiation.

**Figure 5:**
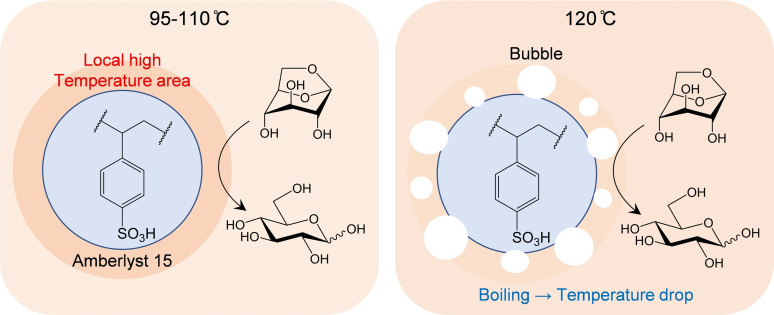
Effect of microwave heating on hydrolysis of levoglucosan.

Figure 5 reveals the reaction behavior in the vicinity of the solid acid catalyst during the hydrolysis of LG under microwave irradiation. Selective heating of the sulfonyl groups increases the solution temperature near the solid acid catalyst to be above the equilibrium temperature. This locally high temperature accelerates the hydrolysis of LG because the catalytic reaction occurs in the vicinity of the catalyst.

The activation energy was greater with microwave heating than with oil bath heating at 95 °C to 110 °C (Figure 3). Within this temperature range, the difference in the hydrolysis rate increased with increasing temperature Figure 3), indicating an increasingly pronounced microwave effect with increasing temperature. This is expressed by the *E*a values (129 kJ/mol for microwave and 103 kJ/mol for oil bath). The relative heating efficiency of water and the sulfonyl group might change with temperature. Horikoshi et al. reported a decreasing efficiency of water heating by microwave irradiation with increasing temperature.^[10]^

**Figure 6:**
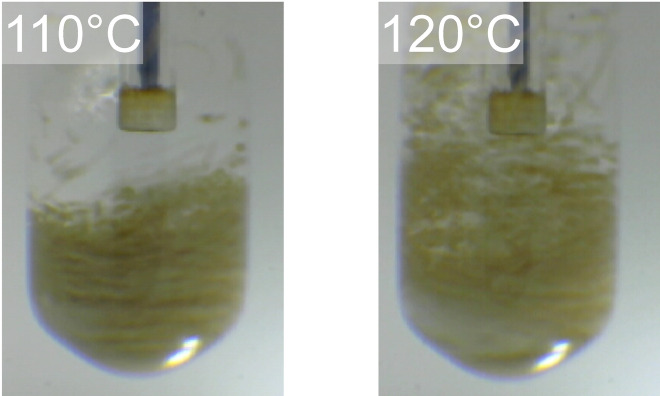
Comparison of appearance under microwave heating.

**Figure 7:**
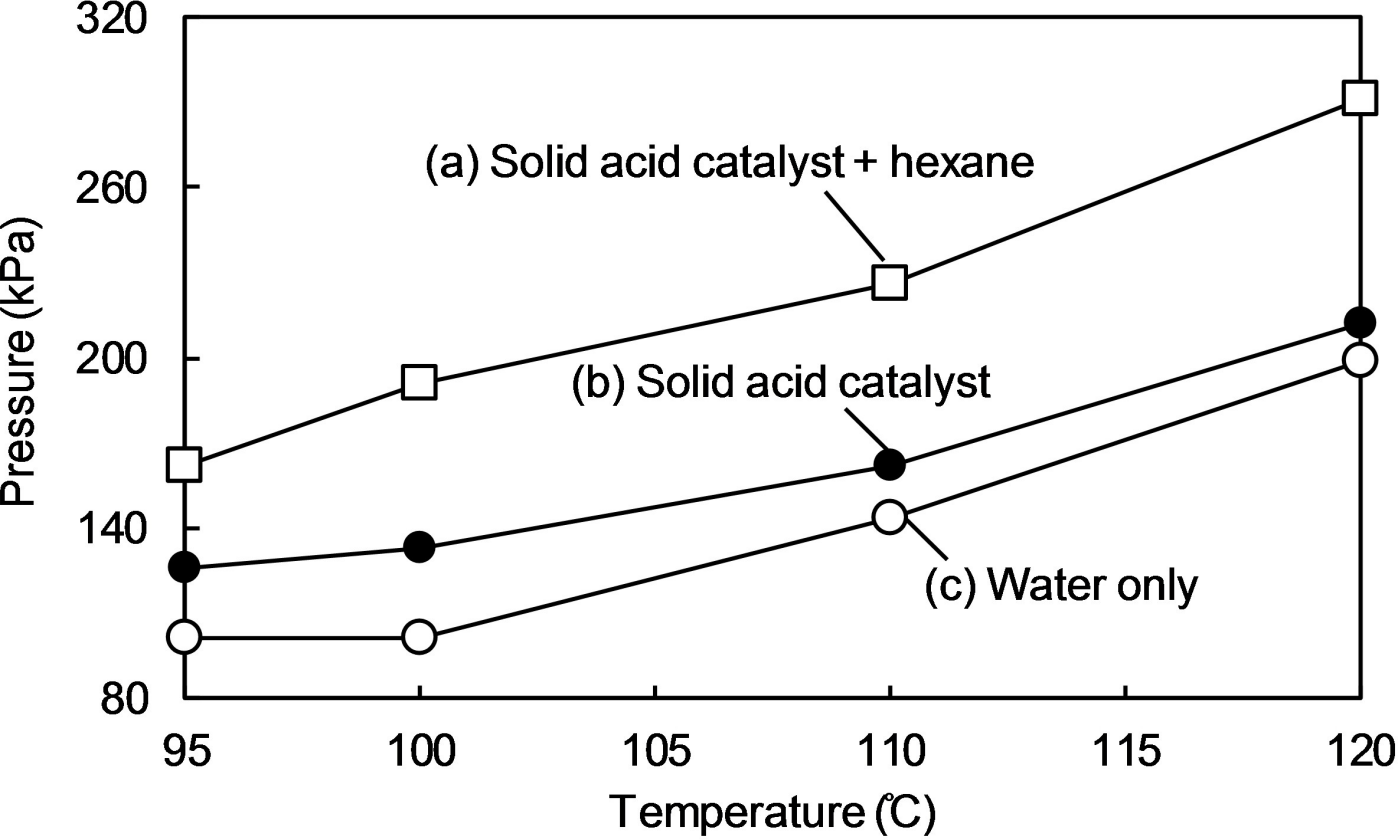
Pressure profiles under microwave heating in (a) solid acid catalyst (Amberlyst 15) and hexane, (b) solid acid catalyst, and (c) water only.

**Figure 8:**
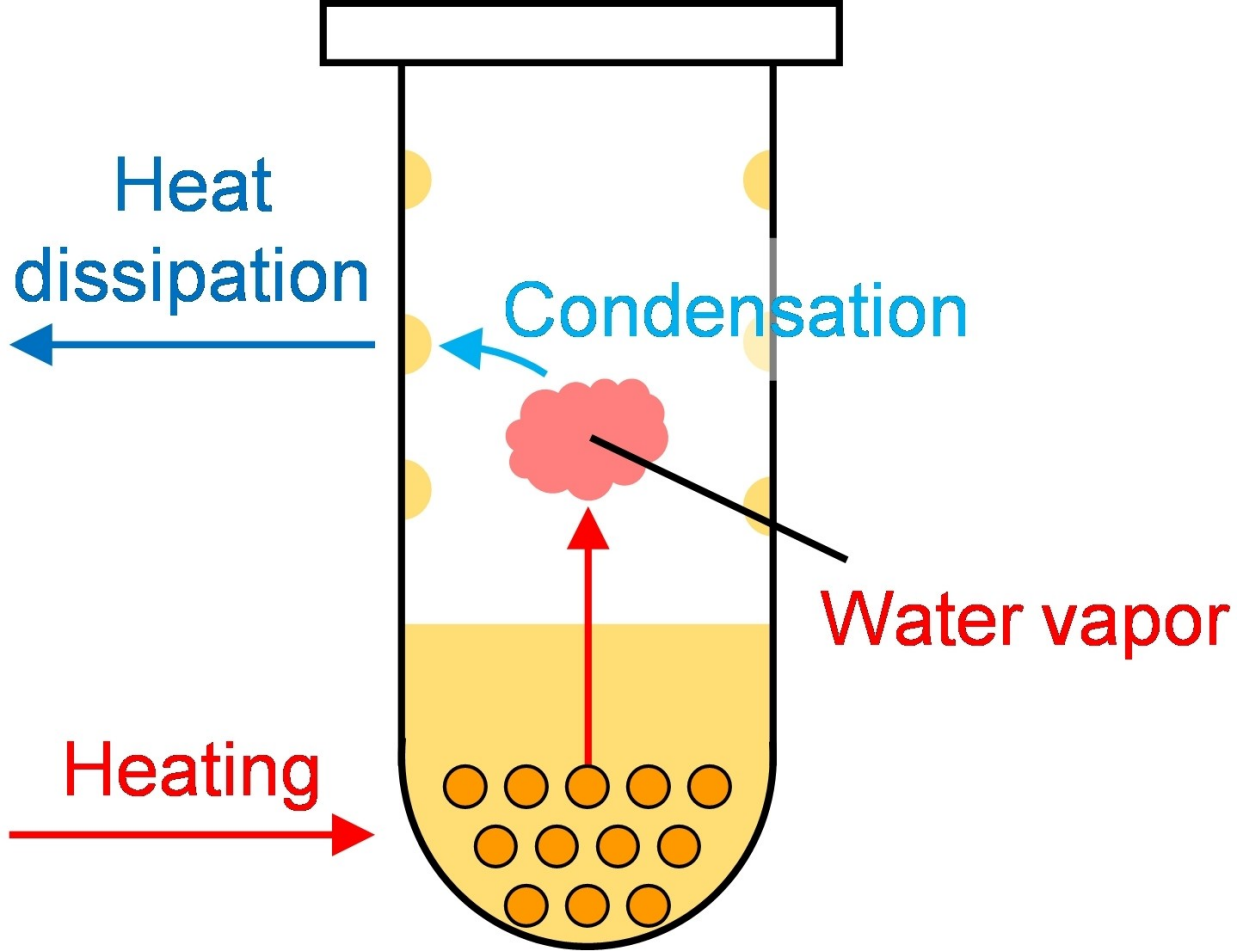
Schematic of the inside of the reaction tube at 120 °C under microwave heating.

At 120 °C, the positive microwave effect on the hydrolysis rate of LG was no longer evident. The solid acid catalyst floated because of boiling of the solution at 120 °C (Figure 6). The difference in the pressure of the solid acid catalyst suspension and water only also decreased with increasing temperature (Figure 7). Figure 8 indicates these observations. In the microwave heating system used in this study, the reaction pressure was increased by the evaporation of water, whereas at 120 °C the water vapor tended to condense onto the upper side of the reactor wall because of the lower wall temperature, resulting in a reflux condition (Figure 6) by maintaining the pressure. As a result, the temperature near the sulfonyl groups was close to the equilibrium temperature. The heating energy was balanced by the energy of water condensation, which was released to the outside of the reactor. This is because of using steam to maintain a high pressure in the closed reactor.

**Figure 9:**
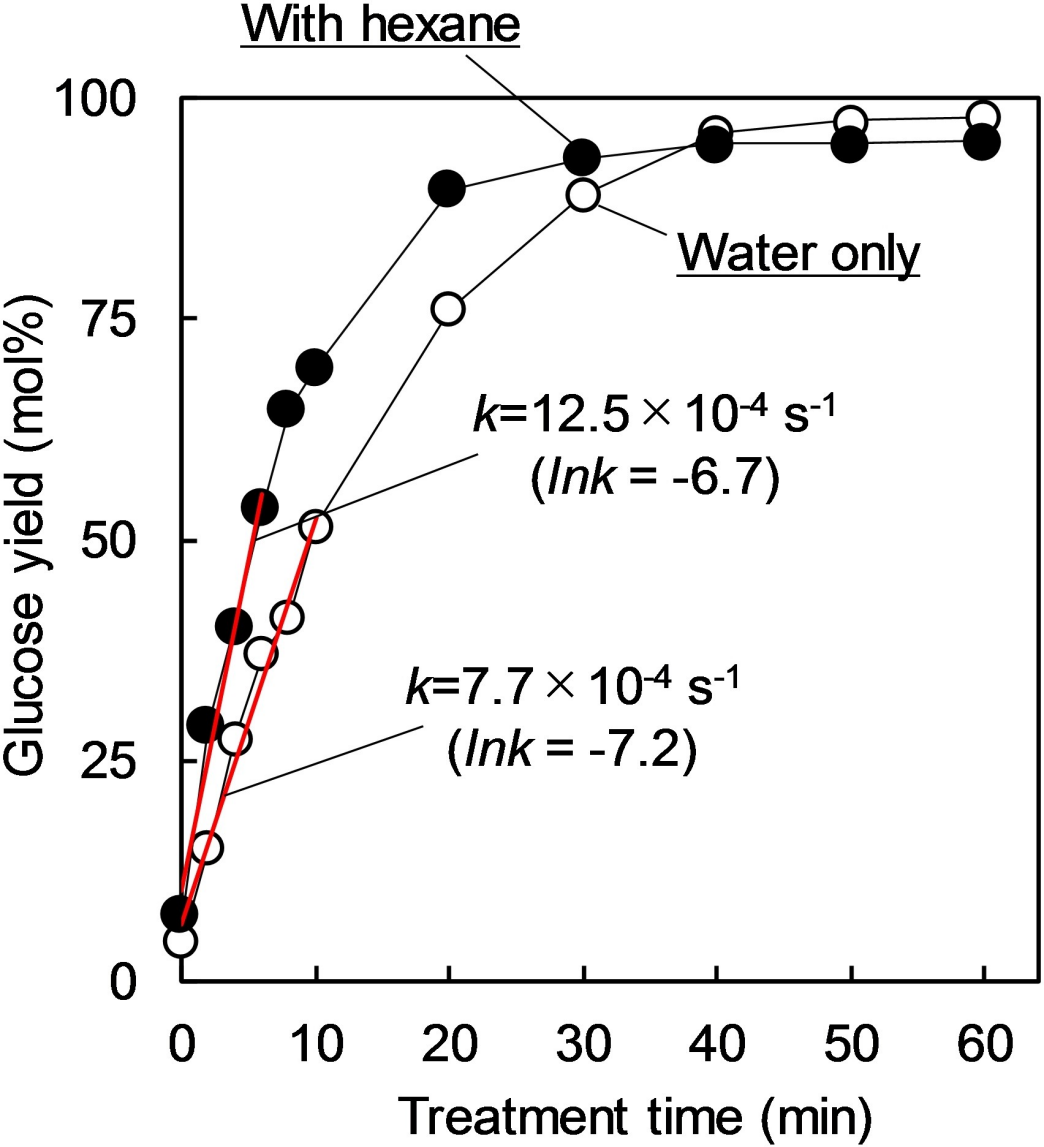
Glucose yield after hydrolysis of levoglucosan in the presence and absence of hexane in water by using a solid acid catalyst (Amberlyst 15) under microwave heating at 120 °C.

To test the aforementioned hypothesis, hexane (0.5 mL) [which has a lower boiling point (69 °C) than water] was added to the reactor. In this system, hexane acted as a gaseous medium that increased the reaction pressure. Figure 7 shows the pressure increase by using hexane. The glucose yield tended to increase by introducing hexane into the hydrolysis of LG at 120 °C by microwave irradiation (Figure 9). The rate constant (ln *k*) increased from −7.2 (water only) to −6.7 (water+hexane) (Figure 3). The boiling temperature of water near the sulfonyl groups of the catalysts increased under such high‐pressure conditions, resulting in a higher local temperature even at the high temperature of 120 °C.

The hydrolysis of LG was also investigated at higher concentrations of 100, 200, and 300 g/L under the reaction conditions of 120 °C for 60 min (Table 1). At all concentrations, the LG conversion was close to 100 mol%. Although the glucose yield tended to decrease slightly as the concentration increased, the yield of glucose was over 90 mol% even at 300 g/L. Therefore, a high‐concentration glucose solution – which is preferable for the subsequent biochemical conversion – can be easily achieved by this method.

**Table 1:** Effect of the levoglucosan (LG) concentration on LG conversion and glucose yield in microwave heating.

	Concentration of LG (g/L)			
	10	100	200	300
LG conversion (mol%)	98.5	98.3	97.7	97.5
Glucose yield (mol%)	98.5	95.4	93.1	90.2

## Conclusions

Hydrolysis of LG by using a solid acid catalyst (Amberlyst15) was performed by microwave heating and the results were compared with those of oil bath heating. LG was almost completely converted into glucose at 120 °C/60 min under both heating conditions up to the concentration of 300 g/L. Hydrolysis was more efficient under microwave heating than in the oil bath under sufficiently high reaction pressure conditions. The sulfonyl groups in the catalyst were selectively heated by microwave absorption, resulting in locally higher temperatures near the catalysts than the equilibrium temperature. This explains the more efficient hydrolysis under microwave irradiation.

## Conflict of Interests

The authors declare no conflict of interest.

1

## Data Availability

The data that support the findings of this study are available from the corresponding author upon reasonable request.
